# Metabolic and Immune Consequences of Antibiotic Related Microbiome Alterations during first-line Tuberculosis Treatment in Bamako, Mali

**DOI:** 10.21203/rs.3.rs-3232670/v1

**Published:** 2023-08-11

**Authors:** Dramane Diallo, Shan Sun, Anou Moise Somboro, Bocar Baya, Amadou Kone, Bassirou Diarra, Mohamed Nantoume, Isaac Koloma, Mahamadou Diakite, Jane Holl, Almoustapha Issiaka Maiga, Moussa Seydi, Grant Theron, Lifang Hou, Anthony Fodor, Mamoudou Maiga

**Affiliations:** University Clinical Research Center (UCRC) of the University of Sciences, Technics and Technologies of Bamako (USTTB); Department of Bioinformatics and Genomics, University of North Carolina at Charlotte, North Carolina; School of Laboratory Medicine and Medical Sciences, University of KwaZulu-Natal, Durban, South Africa; University Clinical Research Center (UCRC) of the University of Sciences, Technics and Technologies of Bamako (USTTB); University Clinical Research Center (UCRC) of the University of Sciences, Technics and Technologies of Bamako (USTTB); University Clinical Research Center (UCRC) of the University of Sciences, Technics and Technologies of Bamako (USTTB); University Clinical Research Center (UCRC) of the University of Sciences, Technics and Technologies of Bamako (USTTB); University Clinical Research Center (UCRC) of the University of Sciences, Technics and Technologies of Bamako (USTTB); University Clinical Research Center (UCRC) of the University of Sciences, Technics and Technologies of Bamako (USTTB); Department of Neurology and Center for Healthcare Delivery Science and Innovation, University of Chicago, Chicago, IL; University Clinical Research Center (UCRC) of the University of Sciences, Technics and Technologies of Bamako (USTTB); Service des Maladies Infectieuses et Tropicales, Fann University Hospital Center, Dakar; Centre of Excellence for Biomedical Tuberculosis Research; South African Medical Research Council Centre for Tuberculosis, Stellenbosch University, Cape Town, Saint Kitts and Nevis; Institute for Global Health, Northwestern University, Chicago, IL; Department of Bioinformatics and Genomics, University of North Carolina at Charlotte, North Carolina; Institute for Global Health, Northwestern University, Chicago, IL

**Keywords:** Tuberculosis, microbiota, drugs, dysbiosis, Bamako, Mali

## Abstract

**Background:**

Tuberculosis (TB) infection is known to lead to the unbalance of the gut microbiota and act synergistically on the decline of the host immune response, when untreated. Moreover, previous work has found a correlation between dysbiosis in the gut microbiota composition and the use of antibiotics. However, there is a need for an in-depth understanding of the metabolic and immune consequences of antibiotic-related microbiome alterations during first-line TB treatment.

**Methods:**

In a longitudinal cohort study, which included TB-infected cohorts and healthy individuals (control group), we studied the anti-TB-related changes in the gut microbiota composition and related functional consequences. Sputum, whole blood and stool samples were collected from participants at four time-points including before (Month-0), during (Month-2), at the end of drug treatment (Month-6) and 9 months after treatment (Month-15). Controls were sampled at inclusion and Month-6. We analyzed the microbiota composition and microbial functional pathways with shotgun metagenomics, analyzed the blood metabolomics using high-performance liquid chromatography (HPLC), and measured the levels of metabolites and cytokines with cytometric bead array.

**Results:**

We found that the gut microbiota of patients infected with TB was different from that of the healthy controls. The gut microbiota became similar to healthy controls after treatment but was still significantly different after 6 months treatment and at the follow up 9 months after treatment. Our data also showed disturbance in the plasma metabolites such as tryptophan and tricarboxylic acids components of patients during TB treatment. Levels of IL-4, IL-6, IL-10, and IFN-γ decreased during treatment and levels were maintained after treatment completion, while IL-17A known to have a strong link with the gut microbiota was highly expressed during treatment period and longer than the 9-month post treatment completion. We found that some fatty acids were negatively correlated with the abundance of taxa. For example, *Roseburia, Megasphaera*, and *alpha proteobacterium* HIMB5 species were negatively correlated (rho = −0.6) with the quinolinate production.

**Conclusion:**

Changes in the composition and function of gut microbiota was observed in TB patients before and after treatment compared to healthy controls. The differences persisted at nine months after treatment completion. Alterations in some bacterial taxa were correlated to the changes in metabolite levels in peripheral blood, thus the altered microbial community might lead to changes in immune status that influence the disease outcome and future resistance to infections.

## INTRODUCTION

Tuberculosis remains ranked as one of the leading causes of death from a single pathogen, *Mycobacterium tuberculosis (MTB)*, with 1.4 million deaths among HIV-negative patients and 187 000 deaths among HIV-positive patients reported only in 2021. One-fourth of the world’s population have been infected by *Mycobacterium tuberculosis* and more than 10 million people develop the disease each year [1]. Multiple underlying environmental conditions, immune, and host genetic predisposing factors have been associated with TB infection, such as HIV infection, diabetes, and deficiency in interferon-gamma (IFN-γ) encoding genes [2, 3]. Furthermore, treatment duration, antibiotics adverse events, and drug resistance are important issues to consider. Standard first-line TB treatment requires six months of combination treatment with isoniazid, rifampicin, ethambutol, and pyrazinamide [4]. However, treated and cured individuals are at least 8 times more likely to experience a new episode of TB disease than the general population [5–7]. Genetics, immune deficiency from HIV infection, and gut microbiota damage (dysbiosis) resulting from the use of large-spectrum antituberculosis drugs such as rifampicin [8] are potential factors contributing to such recurrence.

Disruption of microorganisms, composed of bacteria, archaea, and fungi living in the digestive tracts of humans and animals or gut microbiota, [9] are considered as a key factor that has emerged as potentially contributing to TB reinfection [9, 10]. Several studies investigating disruption of the gut microbiota during TB disease and its treatment, identified a long-lasting dysbiosis for up to two years after treatment completion, but none has addressed the metabolic consequences of the damage [5, 11].

However, there is a gap in knowledge about the functional metabolic consequences of the long-lasting dysbiosis caused by anti-TB treatment. In addition, the gut microbiota is known to produce metabolites, called microbial-linked metabolites such as short-chain fatty acids (SCFAs) and others that contribute to the overall metabolic function of the host including its defense against pathogenic microorganisms and drug metabolism during treatments [12]. This complex network, through cellular receptors, is essential to the signaling paths between the microbiota and its host-involved mechanisms [13, 14]. For instance, anaerobic commensals produce enzymes that degrade dietary fibers into SCFAs (such as acetate, propionate, and butyrate). These gut microbiota-linked metabolites regulate host immune-inflammatory response. In addition, butyrate, a product mainly metabolized by Firmicutes [15], interact with the host through the G-protein coupled receptor 109 alpha (GPR109a) [16].

We conducted a cross-sectional clinical study to investigate the changes of the gut microbiota profiles in TB-infected patients before, during, and after treatment, and in healthy controls, using shotgun metagenomics, metabolomics, and the human Th1/Th2/Th17 cytometric bead array for cytokines measurement. To our knowledge, this study is the first of its kind to use shotgun metagenomics to study the effect of anti-TB treatment on gut microbiota over time. This study may provide new knowledge about potential strategies to improve the TB treatment efficacy, using host microbiota directed-therapies [10].

## MATERIALS AND METHODS

### Study Design and Setting

We conducted a prospective cohort study, from February 2016 to August 2020, enrolling consecutively newly diagnosed adults with sputum smear-positive (confirmed later to be TB culture positive) from local TB diagnostic and treatment centers in Bamako, Mali. Bamako, the capital city, has a population of approximately three million people, about 14% of the population of Mali, living in six urban districts, with each district having a health referral center, where TB diagnostic and treatment services are available. In 2019 alone, more than one third of the total TB patients in Mali (6,902 patients) were diagnosed and managed in Bamako.

The study population included a group of patients and healthy individuals matched by age and gender. The TB-infected participants had four study visits for clinical investigation and sample collection as follows: before TB treatment starts, two months and six months during anti-tuberculosis therapy, and then nine months after treatment completion. However, Healthy individuals’ samples were collected at recruitment and six months after inclusion.

### Study Subjects and Samples Processing

Samples collected include sputum, plasma, and stool from each participant. Participants of 18 years old and more, who were newly diagnosed microscopically with pulmonary tuberculosis (TB Group) and healthy control volunteers, with no TB disease or latent TB as confirmed by the QuantiFERON-TB Gold assay (QFT-Plus; Qiagen, Hilden, Germany) as the control group. All the participants were confirmed HIV seronegative and without prior anti-tuberculosis therapy or antibiotics in the past 4 weeks. A written and signed informed consent form was obtained from each participant before being enrolled. The confirmed cases of the newly diagnosed TB patients were followed before and after starting a first-line antituberculosis treatment regimen comprising two months of isoniazid (H), rifampin (R), pyrazinamide (Z), ethambutol (E), followed by four months of rifampin (R), and isoniazid (H) (2HRZE/4RH).

Shotgun metagenomic sequencing was performed at the University of Illinois at Chicago Research Genome Core Laboratory using the laboratory standards procedure with Illumina. Levels of validated plasma markers of gut microbiota-produced metabolites (metabolomics), including SCFAs, were evaluated using high-performance liquid chromatography (HPLC) by Metabolon. Plasma samples collected from TB patients were tested with a Becton Dickinson (BD) LSR II flow cytometer to measure the level of cytokines.

### Mycobacterial identifications

Early morning sputum specimens collected from presumptive TB patients were tested for TB using the standard N-acetyl-L-cysteine/4% sodium hydroxide solution for sputum digestion and decontamination, thereafter, the sample was concentrated by high-speed centrifugation. The pellets were used to inoculate liquid medium (Mycobacterium Growth Incubator Tube (MGIT^™^) [BD, Sparks, MD, USA], and solid medium (Middlebrook 7H11 agar and selective 7H11 agar) to isolate pure mycobacteria colonies, as previously described [17]. The concentrated specimen also served simultaneously to prepare an auramine-rhodamine smear for fluorescent acid-fast microscopy visualization (BBL^™^, BD, Sparks, MD, USA). The speciation of the isolated mycobacteria was based on the observation of colonial morphology from culture media and the use of Gen-Probe TB molecular assay (AccuProbe^®^, San Diego, CA, USA) [18].

### Cytokine Measurements

Cytokines’ levels over time were determined to evaluate the immune evolution of the disease from pretreatment to post-treatment status. To achieve this, the Human Th1/Th2/Th17 kit was used according to the manufacturer’s instructions (BD Biosciences, Torreyana Rd, San Diego, USA). The BD Cytometry Bead Array (CBA) was used to evaluate the following cytokines levels: Interleukin-2 (IL2), Interleukin-4 (IL4), Interleukin-6 (IL6), Interleukin-10 (IL10), Tumor Necrosis Factor (TNF), Interferon-gamma (IFN-γ) and Interleukin-17 alpha (IL17A) from plasma samples.

Briefly, before running the assays, samples were removed from −80°C to the cold room (4-8°C) for thawing. Samples and standards were then prepared and measured following the manufacturer’s instructions, by using an LSR II flow cytometer at the University Clinical Research Center (UCRC) immunology core facility laboratory. Analysis was then performed by FCAP Array ^™^ software version 3.0.1. The detection limits for each cytokines measured were as follow: IL-2 (2.6 pg/mL), IL-4 (4.9 pg/mL), IL-6 (2.4 pg/mL), IL-10 (4.5 pg/mL), TNF (3.8 pg/mL), IFN-γ (3.7 pg/mL) and IL-17A (18.9 pg/mL).

### DNA Extraction from stool samples

DNA was extracted from stool samples using the QIAmp DNA Stool Mini Kit (Qiagen, Hilden, Germany). Frozen stool samples were placed and tawed on ice before the extraction starts. The stool samples were first weighed (180-220 mg of thawed stool) then extraction was performed according to the manufacturer’s instructions. The extracted DNA was then measured using the Nanodrop One^c^ (Thermofisher Scientific, Verona Rd, Madison, USA) to assess the DNA concentration and its purity, before sending for sequencing.

### Shotgun metagenomics of the gut microbiome and bioinformatics analysis

DNA extracted from stool samples was sequenced at the University of Illinois at Chicago (UIC) Research Genome Core Laboratory using Illumina HiSeq, and the sequences were analyzed by Dr. Anthony Fodor’s team at the University of North Carolina at Charlotte. The host contamination of shotgun metagenomics reads was removed using Kneaddata. The taxonomic composition of the microbiome was characterized using Kraken2, and the functional pathways (stratified pathways and unstratified pathways) were characterized using HUMAnN2 following the developers’ instructions. The R software was used for downstream statistical analysis of taxonomic composition and pathway abundances. The PCoA ordination was calculated with function ‘capscale’ in the R package ‘vegan’ using Bray-Curtis dissimilarity. The Shannon diversity was calculated with function ‘diversity’ in the same package. The linear mixed-effects models were performed with function ‘lme’ in R package ‘nlme’, with healthy/TB group, timepoints, and their interaction as the main effects and subject ID as the random effects. P values were adjusted using the Benjamini-Hochberg method to correct for multiple hypotheses testing. False Discovery Rate (FDR) <0.05 was considered as statistically significant.

### Metabolomics Analysis of plasma samples

Metabolomic analysis was performed on the plasma samples collected from TB infected individuals (including samples before treatment, at 6-month treatment, and at 9-month after treatment completion). Each sample received was accessioned to the Metabolon Laboratory Information Management System (LIMS) and was assigned by the LIMS a unique identifier that was associated with the source identifier only. This identifier was used to track all sample handling, tasks, results, etc. The samples (and all derived aliquots) were tracked by the LIMS system. All portions of any sample were automatically assigned their unique identifiers by the LIMS when a new task was created; the relationship of these samples was also tracked.-

Plasma samples were prepared using the automated MicroLab STAR^®^ system from Hamilton Company. Several recovery standards were added before the first step in the extraction process for QC purposes. To remove protein, dissociate small molecules bound to protein or trapped in the precipitated protein matrix, and recover chemically diverse metabolites, proteins were precipitated with methanol under vigorous shaking for 2 min (Glen Mills GenoGrinder 2000) followed by centrifugation. The resulting extract was divided into five fractions: two for analysis by two separate reverse phases (RP)/UPLC-MS/MS methods with positive ion mode electrospray ionization (ESI), one for analysis by RP/UPLC-MS/MS with negative ion mode ESI, one for analysis by HILIC/UPLC-MS/MS with negative ion mode ESI, and one sample was reserved for backup. Samples were placed briefly on a TurboVap^®^ (Zymark) to remove the organic solvent. The sample extracts were stored overnight under nitrogen before preparation for analysis. The metabolomics results were analyzed using R software. GraphPad prism version 8.0.1, was used for patients’ social characteristics analyses and cytokines measurement analysis.

### Availability of data and materials

Not available.

## RESULTS

### Participants’ Socio-demographic and Clinical Characteristics

The longitudinal cohort study involved thirty-two (32) confirmed TB individuals and twenty-seven (27) healthy controls (TB and HIV negative). The TB-infected participants were followed up for over 15 months including six months of the treatment and then nine months after the treatment completion, whereas the healthy control individuals were followed up for six months to look for the gut microbiome’s natural evolution. Most of the participants were men, with 25/30 (83.33%) for TB-infected and 17/26 (65.38%) for healthy individuals. The mean age was 32.53 years old [18-72 years] for TB-infected, while for healthy controls it was 25.8 [18-39]. About 29% of all participants were smokers, with 11/32 (34.37%) for TB patients, and 6/27 (22.22%) for healthy individuals. A total of 24/32 (75%) of the TB patients were infected with the modern Euro-American lineage 4, which showed a high amount of persistence of sputum smear positivity at month-2 of TB treatment 8/10 (80%). The other circulating lineages in Mali, lineage 1, lineage 2 and lineage 3 were more responsive to the anti-TB drugs, 10/12 (83.33%) at this time point. A total of 4/10 (40%) of the persistent cases at two months of treatment were observed among smoking participants.

### Longitudinal Metagenomics Analysis of Microbial Diversity in the Gut of TB patients during drug treatment

**Figure 1:**
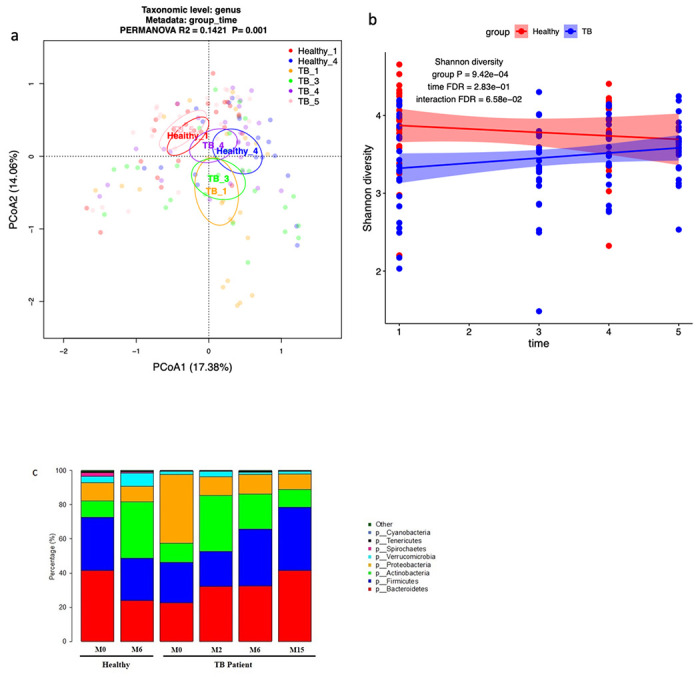
Gut microbiota diversity and taxonomic composition of TB patients and healthy controls.

The Principal Coordinate Analysis (PCoA) at the genus level showed the separation of gut microbial communities by study groups. For longitudinal analysis purposes, the TB-infected individuals are shown here by time-points, which include TB 1 (TB patients prior to initiation of TB treatment), TB 3 (2 months after initiation of TB treatment), TB 4 (6 months after initiation of TB treatment), TB 5 (9 months after completion of TB treatment). The healthy control group is named, Healthy 1 (healthy individuals at inclusion into the study) and Healthy 4 (6 months after study inclusion). The gut microbiota of TB patients before treatment (TB 1) and after 2-month treatment (TB 3) are separated from that of healthy controls. The gut microbiome composition of TB patients changed over time during and after treatment, with TB 4 and 5 groups more similar to healthy controls compared to TB 1 and 3 (Figure 1a). However, TB 4 and 5 were still significantly different from healthy controls with PERMANOVA tests (TB 4 vs Healthy 4: R^2^=0.059, P=0.004; TB 5 vs Healthy 4: R^2^=0.015, P=0.001). In addition, healthy groups were also separate (different from each other) in a six month sampling period showing microbiota instability. The Shannon diversity of the gut microbiota of TB patients increased with time and are not significantly different from that of health controls 6 month after treatment (Figure 1b). In addition, we observed increased relative abundance of Bacteroidetes and Firmicutes after treatment and their abundance increased more at 9 months after the completion of TB treatment (Figure 1c). In contrast, the relative abundance of inflammation related Proteobacteria decreased after treatment. In general, the gut microbiota was altered in TB infected patients compared to the healthy controls, but the differences decreased after treatment at the 6-month timepoint. However, the microbial communities were still different from healthy controls after treatment.

**Figure 2:**
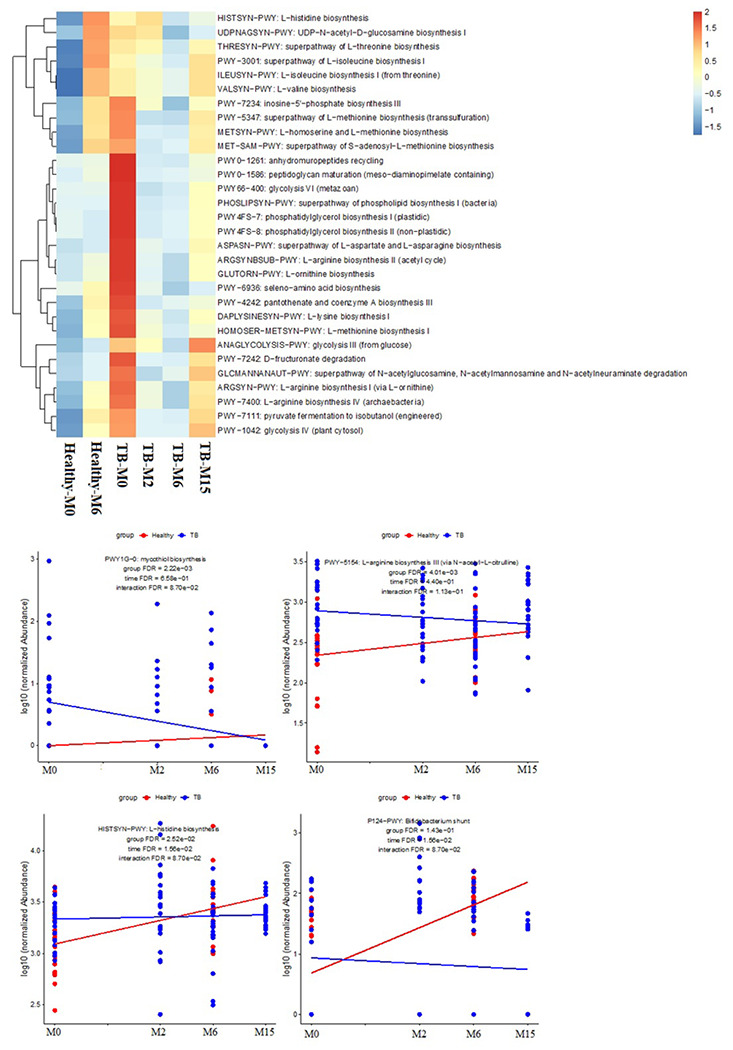
Differences in gut microbial functional pathways between TB patients and healthy subjects. Pathways shown in the heatmap are significantly different between TB patients and healthy controls with mixed effects linear models adjusted for timepoints and the interaction between group and timepoints (see methods). Pathway with lower abundance (<0.02%) are excluded from the analyses. Keys indicate z-scores of relative abundances. Scatter plots showed the changes of four significant functional pathways.

This study revealed that the microbial functional pathways were also altered by TB infection and its treatment. The microbial functional pathway profiles of TB patients before treatment were distinct from the healthy controls, while those after treatment are more like the controls. An increase of amino acids and lipids biosynthesis pathways was observed in TB groups compared to healthy controls. In contrast, the glycosis III and IV biosynthesis pathways, Arginine I and IV biosynthesis pathways, pyruvate fermentation to isobutanol pathway, D-fructuronate degradation pathways and the super pathway of N-acetylglucosamine, N-acetylmannosamine and N-acetylneuraminate degradations were found to be upregulated while the selenoamino acids biosynthesis pathway is down regulated. Some of the pathways such as lipids, glycosis, and aminoacids biosynthesis pathways remained altered even nine months after treatment completion suggesting long-lasting dysregulated effects after TB treatment (Figure 2).

### Metabolomics analysis

After metabolomics analysis, 472 biochemicals were found to be statistically significant (P≤0.05) and 72 approaching significances (0.05<P<0.10). Three groups are compared to evaluate the changes of blood metabolites, before the 6-month treatment (pretreatment/pre-standard), at the completion of the treatment (standard), and 9-month after treatment completion (post standard) (Table 1).

**Figure 3:**
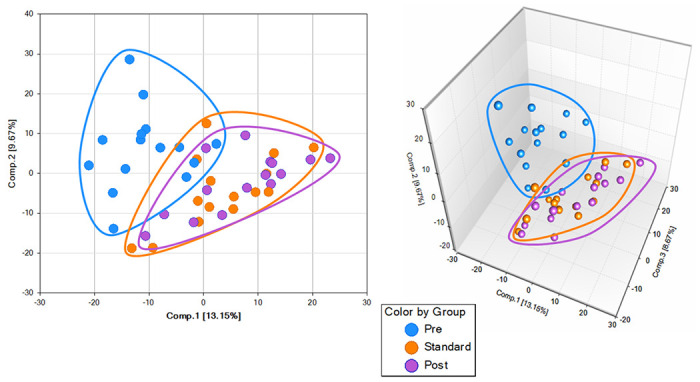
Principal component analysis showing separation of the different groups, before, treatment, six-month of treatment, and nine-month after treatment. Data are displayed in two dimensions (**A**) and three dimensions (**B**).

Principal Components Analysis (PCA) plots showed that the pre-treatment time-point is distinct and separated from the standard treatment completion and the post-treatment timepoint indicating that the treatment effects on metabolites were maintained at least nine months after treatment completion (Figure 3).

**Figure 4:**
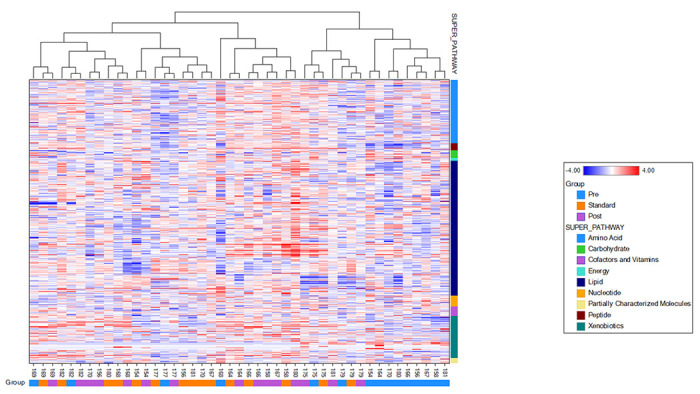
Hierarchical clustering analysis of metabolites pathways from plasma

The Standard-treatment and post-treatment overlap, suggesting that their metabolic profiles are similar and different from the pre-treatment/infection stage. Using the Hierarchical Clustering Analysis (HCA), we found 5 grouping of 15 subjects’ samples of metabolic profiles (Figure 4). There is also a cluster of 9 pretreatment samples, suggesting good similarities among many pre-treatment samples. The lack of such high clustering at the end of the standard treatment and nine months after treatment suggested that the variance from these two timepoints are smaller than the variance across the study subjects.

**Figure 5:**
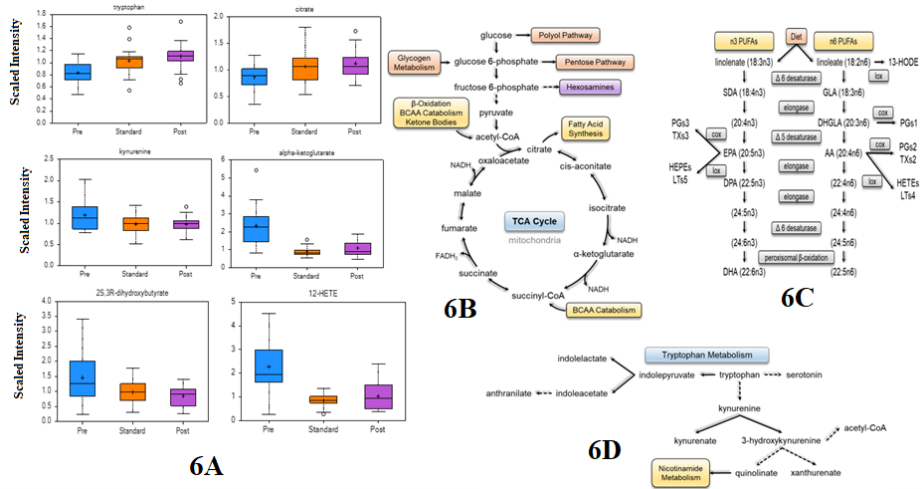
Main biochemical pathways altered during, and after TB disease

Our data showed major changes between TB timepoints in four main pathways as reported in Figure 5, such as tryptophan metabolism, fatty acid metabolism, and energy pathways.

### Correlation between genus and pathways abundance and produced microbial metabolites.

**Figure 6:**
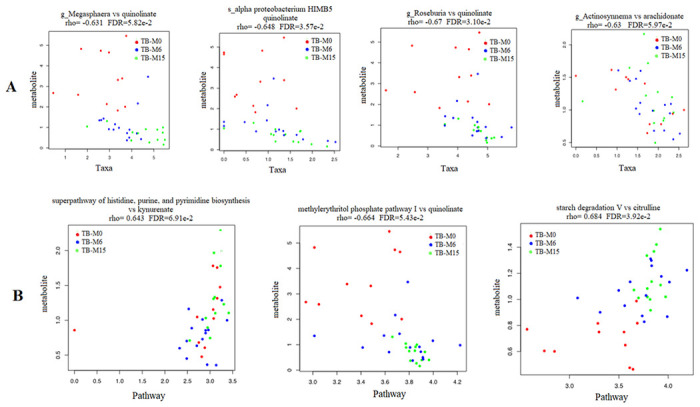
Correlation between microbial-produced metabolites and both pathways and taxa abundance **A** Quinolinate was negatively correlated with the abundance of genera Megasphaera, Roseburia and the species *Alpha proteobacterium HIMB5* from the gut microbiota. Arachidonate was negatively correlated with the abundance of genus Actinosynnema. **B.** Citrulline and kynurenate were found to be positively correlated with starch degradation V and histidine, purine and pyrimidine biosynthesis pathways respectively, while the quinolinate was negatively correlated with methylerythritol phosphate pathway I.

Our study found significant correlations between genus/pathway and metabolite abundance in the gut during the six-month TB antibiotic regimen. Using the Spearman correlation test, we compared the metabolites’ production with thetaxa’s relative abundance at six-month of treatment and nine months after treatment completion (Figure 6A). The whole correlated taxa from gut microbiota to peripheral metabolites are listed in **supplemental data 1.** The taxa correlated with metabolites production belong to the bacteria phyla of Proteobacteria, Actinobacteria, and Firmicutes, and Euryarchaeota from the archaea domain. The fatty acids’ such as quinolinate and arachidonate levels that are important for the inflammatory balance/pathway, were found to decrease with the increase of these taxa. In addition, we found correlations between the pathways’ alterations and the metabolites produced (Figure 6B). A negative correlation was found between quinolinate and both Guanosine and methylerythritol phosphate pathway I. The ornithine was also found to negatively correlate with the alteration of glycolysis, tricarboxylic acid cycle, and glyoxylate bypass. In addition, citrulline and kynurenate were found to be positively correlated with the alteration of histidine, purine and pyrimidine biosynthesis (kynurenate), aromatic amino acid biosynthesis and starch degradation V pathway (citrulline) in the samples of TB patients.

### Cytokines Analysis with CBA

**Figure 7:**
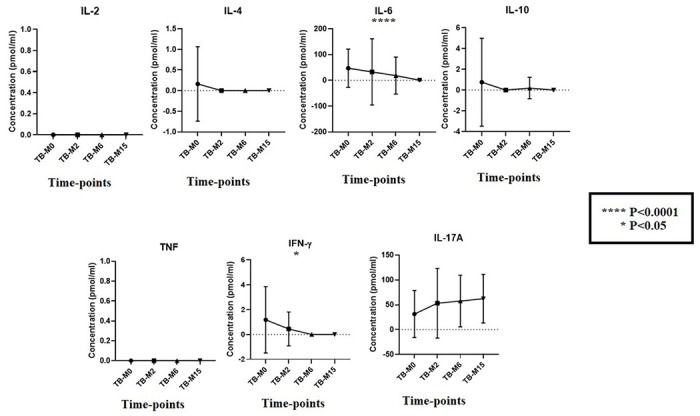
Cytokines production at study time points for TB groups

The immunological profile of participants was analyzed with the cytokines levels that are relevant from the literature for both TB disease and the microbiome (Figure 7). We found that the mean cytokines levels were high for the major inflammatory players that are important for TB disease, such as IL-4, IL-6, IL-10, and IFN-γ before treatment started **(TB-M0),** but the levels continue to decrease slowly during and after treatment completion. In contrast, IL-17A known to have a strong link with the gut microbiota was highly expressed during the treatment period and the trend was maintained a long time after completion **(TB-M15)**.

## DISCUSSION

This study revealed that the gut microbiome of TB patients before and after treatment were different from that of healthy controls; the altered microbial community in the gut environment persisted for at least nine months after treatment completion. The gut microbiota is involved in the biological homeostasis of the host by its implication in the production of molecules that interact with the host cells. The dysregulation likely due to the *M. tuberculosis* infection tends to become normal after the inflammation storm caused by the infection itself. Previous studies mentioned this diversity related to TB infection during and after treatment [5,19,20]. However, we found herein that the use of anti-mycobacterial drugs is also associated with disrupting human gut microbes. Thus, the gut microbiome dysbiosis induced by anti-tuberculosis treatment may contribute to the risk of recurrent TB. This hypothesis was supported by previous findings about treated TB-infected individuals having higher chances of developing a new episode of TB infection as compared to untreated [7,21,22]. This could be explained by the long-lasting damage from TB drugs on the gut microbiota and the resulting impact of its homeostatic role in inflammation and other metabolic functions that are essential for resistance against TB.

Recent studies showed significant relationships between changes in the gut microbiota and many human disease outcomes, including tuberculosis [23–26]. We conducted here a longitudinal study to monitor changes in the gut microbiota’s composition and relevant microbial-linked metabolic pathways in TB-infected individuals in comparison with healthy control individuals. Men represented the majority of TB-infected as reported in several other studies [27–29]. The mean age for TB patients was 32.5 and the smoking status represented 33%, studies on TB revealed its impact in young men and its association with cigarette use [11,30,31].

Despite the high rate of bacteria in gut microbiota composition, other species such as Archaea, viruses, and fungi were impacted during treatment but are generally less investigated than Bacteria. Our data showed significant differences in the Euryarchaeota phylum from the archaea domain. One of the two major known archaeal phyla, Euryarchaeota decreased from the gut microbiota of TB patients before treatment and at 2-month of treatment (with isoniazid and rifampicin regimen), then the level increase at the end of therapy before being finally decreased at nine months after treatment completion.

As reported by Negi S *et al* in mice model, the use of broad-spectrum TB antibiotics such as rifampicin active on gram positive bacteria, could contribute to changes in the gut microbiota diversity and composition [32]. In fact, the persistence of the alteration lasted nine months after treatment completion. Our data showed a negative correlation between the genus Actinosynnema, Megasphaera, and Roseburia relative abundances and the quinolinate metabolite. Quinolinate (quinolinic acid) is known as a marker for kynurenine in the tryptophan pathway [33]. Similarly, Shibata *et al.* also reported disturbing effects after administration of antituberculosis drugs (mainly pyrazinamide and isoniazid) on the metabolic pathways of quinolinic acid [34]. Furthermore, the kynurenine/tryptophan ratio is reported to be a great biomarker for pulmonary tuberculosis [35]. Thus, it seems that the dysbiosis of the above genus was caused by antibiotics during TB treatment leading to plasma tryptophan pathway alteration. On other hands, the arachidonic acid (arachidonate), a polyunsaturated fatty acid was found in our study to negatively correlates with the abundance of the genus of Actinosynnema. It’s known to be one of the preferred nutrients of *M.tb* through the biosynthesis by infected macrophages and impair their inflammatory and antimicrobial activities [36]. Our data showed the persistence of this polyunsaturated fatty acid nine months after the end of TB treatment, which could additionally explain the vulnerability of treated and cured TB patients to new episodes of tuberculosis disease as compared to the general population, up to 8-fold [36].

We measure the level of seven cytokines from the plasma of TB patients and compare the level between groups. IL-6 and IFN-γ were found to be more significant, which play important role in the acute phase response against TB [37,38]. During the treatment, we saw the decrease of their levels before the total bacterial clearance. The negative effect of antibiotics on the microorganism responsible of immunomodulator metabolites.

This study has some limitations: The sample size could be bigger to established strongly these important new findings, although the numbers used in this study are similar to other similar studies [26,34]. Future studies will include many different populations with a bigger sample size to generalize the findings. In addition, the effects of the TB drugs were measured collectively together and not individually. It is not ethical to treat TB patients with single drug, but we did this in animal model in the past and found that rifampin, a large spectrum antibiotic was responsible for the majority of dysbiosis seen with this drug regimen [5]. Nevertheless, this study is the most comprehensive analysis of the consequences of TB drug related dysbiosis during and after treatment and will advance the field of TB microbiome and our understanding of involved mechanisms.

## CONCLUSION

This study shows the persistence of the gut microbiota dysbiosis caused by antituberculosis drugs up to nine months after treatment completion. Furthermore, dysbiosis impacts the microbial-linked metabolites and their pathways, which contribute to weakening the inflammatory balance in TB-treated participants and make them vulnerable to another episode of TB disease. Additional studies are needed to verify the main findings of this study. The findings have major implications in the use of host microbiota-directed therapies and the development of TB therapies that are more friendly to the gut microbiota, such as rifapentine, to preserve its essential functions during and after treatments.

## Figures and Tables

**Table 1:** Statistical Comparison of the plasma metabolites

ANOVA Contrasts	Month-6 versus Month-0	Month-15 versus Month-0	Month-15 versus Month-6
Total biochemicals (P≤0.05)	391	442	179
Biochemicals (+ −)	238/153	285/157	103/76
Total biochemicals (0.05<P<0.1)	76	93	69
Biochemicals (+ −)	47/29	66/27	41/28

Numbers in red indicate the up expressed and green, the down expressed molecules
